# Computational identification of promoters in *Klebsiella aerogenes* by using support vector machine

**DOI:** 10.3389/fmicb.2023.1200678

**Published:** 2023-05-05

**Authors:** Yan Lin, Meili Sun, Junjie Zhang, Mingyan Li, Keli Yang, Chengyan Wu, Hasan Zulfiqar, Hongyan Lai

**Affiliations:** ^1^Key Laboratory for Animal Disease-Resistance Nutrition of the Ministry of Agriculture, Animal Nutrition Institute, Sichuan Agricultural University, Chengdu, China; ^2^Beidahuang Industry Group General Hospital, Harbin, China; ^3^Chifeng Product Quality Inspection and Testing Centre, Chifeng, China; ^4^Nonlinear Research Institute, Baoji University of Arts and Sciences, Baoji, China; ^5^Baotou Teacher’s College, Inner Mongolia University of Science and Technology, Baotou, China; ^6^Yangtze Delta Region Institute (Huzhou), University of Electronic Science and Technology of China, Huzhou, Zhejiang, China; ^7^Chongqing Key Laboratory of Big Data for Bio Intelligence, Chongqing University of Posts and Telecommunications, Chongqing, China

**Keywords:** promoter, pseudo k-tuple nucleotide composition, position-correlation scoring function, feature selection, support vector machine

## Abstract

Promoters are the basic functional cis-elements to which RNA polymerase binds to initiate the process of gene transcription. Comprehensive understanding gene expression and regulation depends on the precise identification of promoters, as they are the most important component of gene expression. This study aimed to develop a machine learning-based model to predict promoters in *Klebsiella aerogenes* (*K. aerogenes*). In the prediction model, the promoter sequences in *K. aerogenes* genome were encoded by pseudo *k*-tuple nucleotide composition (PseKNC) and position-correlation scoring function (PCSF). Numerical features were obtained and then optimized using mRMR by combining with support vector machine (SVM) and 5-fold cross-validation (CV). Subsequently, these optimized features were inputted into SVM-based classifier to discriminate promoter sequences from non-promoter sequences in *K. aerogenes*. Results of 10-fold CV showed that the model could yield the overall accuracy of 96.0% and the area under the ROC curve (AUC) of 0.990. We hope that this model will provide help for the study of promoter and gene regulation in *K. aerogenes*.

## 1. Introduction

*Klebsiella aerogenes* (*K. aerogenes*) is a ubiquitous Gram-negative bacterium found in a variety of environments, such as soil, sewage, mammalian gastrointestinal tract et al. The *K. aerogenes* can also colonize in human gut and most community-or hospital-acquired bloodstream infections are caused by this common multi-drug resistant pathogen, which is a source of opportunistic infections. Although most of these bacteria are sensitive to the antibiotics targeting them, the drug resistance still exists, and the induced resistance mechanisms are complex (Price and Sleigh, 1970). Promoters are the genomic regions upstream of genes, where RNA polymerase and other transcription factors bind together to initiate genes transcription (Sawadogo and Roeder, 1985). Thus, promoter identification is the first step to understand gene expression mechanism. Thus, a precise identification of promoter sequence could generate dynamic signs for understanding its mechanism of regulation (Zuo and Li, 2010).

In fact, several experimental methods, such as mass spectrometry (Flusberg et al., 2010), reduced-representation bisulfite sequencing (Doherty and Couldrey, 2014), and single-molecule real-time sequencing (Boch and Bonas, 2010), have been developed to recognize promoters. Although these methods are relatively helpful in the identification of promoters, they are exorbitant when implemented to large sequencing data (Hu et al., 2022a). Therefore, a bioinformatics tool to identify promoter sequence is instantly needed.

At present, some machine learning-based methods have been presented to predict promoters in multiple species (Ao et al., 2022). Li and Lin have ever designed a position weight matrix (PWM) method to identify sigma70 promoters in *Escherichia coli* (*E. coli*) (Li and Lin, 2006). Subsequently, they developed a hybrid approach (called IPMD) to identify eukaryotic and prokaryotic promoters (Lin and Li, 2011). PePPER is another webserver for recognizing prokaryote promoter elements and regulons (de Jong et al., 2012). In 2014, Lin et al. proposed a first model called iPro54-PseKNC to predict sigma54 promoters in prokaryotes (Lin et al., 2014). Liu et al. established a friendly tool called iPromoter-2 l for the prediction of bacterial promotors. These works mainly used sequence composition to perform prediction. By using Z-curve theory, the bacterial promoters could also be formulated and predicted (Song, 2012; Lin et al., 2019). Combining various of sequence information, Lai et al. built a powerful model named iProEP for the identification of promoters in three kinds of eukaryotes and two kinds of bacteria (Lai et al., 2019). Chevez-Guardado designed a general tool (Promotech) for bacterial promoter recognition (Chevez-Guardado and Peña-Castillo, 2021). Recently, the promoters in two prokaryotes: *Corynebacterium glutamicum* and *Agrobacterium Tumefaciens Strain C58* were studied by using machine learning based models (Zulfiqar et al., 1011; Li et al., 2023). Among them, the sigma70 promoter is the most extensively studied in prokaryotes (Patiyal et al., 2022). iProm-phage is a two-layer model for phage promoters and their types prediction (Shujaat et al., 2022).

**Figure 1:**
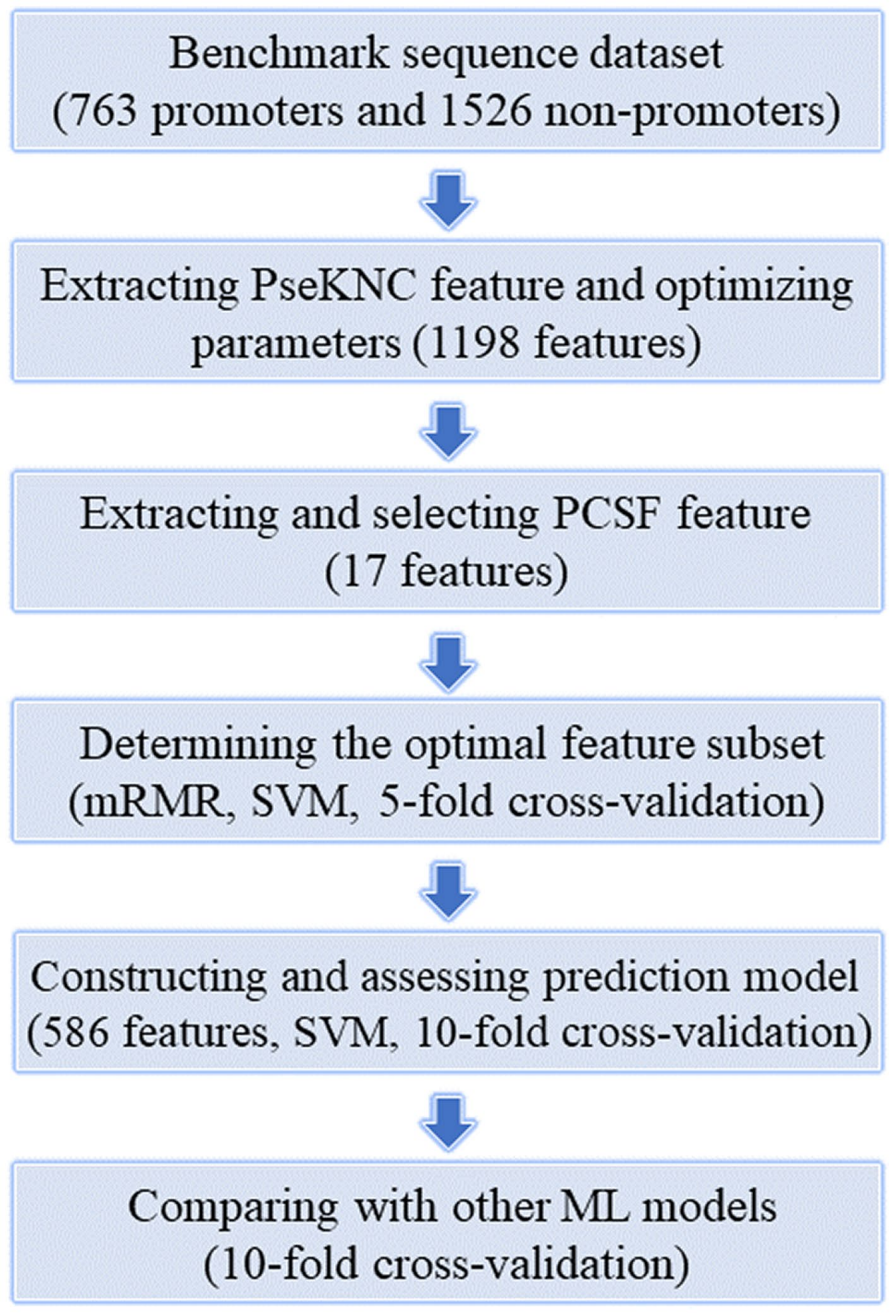
The overall workflow of this study.

Although there are already many prediction models for prokaryotic promoters, due to species specificity and prediction performance limitations, there is a need for trainning more specific promoter prediction models for *K. aerogenes* (Hu et al., 2022b). Thus, in this paper, we designed a SVM-based model to predict the promoters of *K. aerogenes*. The Figure 1 illustrates the workflow of this project, mainly including the core content and key steps. Thereinto, two feature extraction methods, namely PseKNC and PCSF, were employed to convert DNA sequences into numerical features. And then these features were optimized by using mRMR feature selection algorithm based on SVM machine learning model and 5-fold CV. Moreover, the selected optimal feature subset was applied to train a SVM classifier for identifying *K. aerogenes* promoter sequences on the basis of 10-fold CV. As a result, an ideal model with prediction accuracy and AUC of 96.0% and 0.990 was attained.

## 2. Materials and methods

### 2.1. Data collection and preprocessing

The construction of a prokaryotic promoter dataset is crucial for obtaining a good promoter model. Prokaryotic Promoter Database (PDD, http://lin-group.cn/database/ppd/) developed by Lin et al. contains comprehensive information on experimentally verified promoters of numerous prokaryotic species and can be freely accessed (Su et al., 2021). The sequence data of 763 *K. aerogenes* promoters were downloaded from the database and defined as positive dataset. Each promoter sequence was composed of 81 nucleotides, including transcription start site (TSS) (namely the 0-th site), upstream 20 bp and downstream 60 bp regions of TSS. In order to generate a reliable negative dataset, we firstly extracted the convergent intergenic (length greater than 81 bp) and coding (length greater than 2000 bp) regions from *K. aerogenes* genome. Secondly, sliding window method with step of 1 bp was applied to generate convergent intergenic and coding sequences, with length of 81 bp. Then, we used CD-HIT program to estimate the sequence similarity of convergent intergenic and coding sequences, and filtered highly similar sequences by setting cutoff value as 0.8. Finally, 763 convergent intergenic sequences and 763 coding sequences were randomly picked out and regarded as negative dataset.

### 2.2. Feature extraction

Referring to the well-designed eukaryotic and prokaryotic promoter identification tool, iProEP,^1^ we also adopted two algorithms, including pseudo k-tuple nucleotide composition (PseKNC) and position-correlation scoring function (PCSF), to transform raw promoter/non-promoter sequence data into suitable numeric features for modeling.

In this study, the type II PseKNC method was used to transform each nucleotide sequence into a feature vector of $4^{k}+\lambda \Lambda$ dimensions (Tang et al., 2021),


(1) $$D_{pseKNC}={\left[d_{1}\;d_{2}\cdot \cdot \cdot d_{4^{k}}\;d_{4^{k}+1}\cdot \cdot \cdot d_{4^{k}+\lambda }\;d_{4^{k}+\lambda +1}\cdot \cdot \cdot d_{4^{k}+\lambda \Lambda }\right]}^{T}$$

where k means k-tuple nucleotide component, λ is an integer less than L−k (L denotes the length of a DNA sequence). And Λ is the number of physicochemical properties, the value of which is 6 corresponding to the six types of DNA local structural properties included in this work. Each element in $D_{pseKNC}$ is defines as:

(2) $$d_{u}=\left\{\begin{array}{c} \frac{f_{u}^{k-tuple}}{\sum_{i=1}^{4^{k}}f_{i}^{k-tuple}+\omega \sum_{j=1}^{\lambda \Lambda }\tau_{j}},\left(1\leq u\leq 4^{k}\right)\\ \frac{\omega \tau_{u-4^{k}}}{\sum_{i=1}^{4^{k}}f_{i}^{k-tuple}+\omega \sum_{j=1}^{\lambda \Lambda }\tau_{j}},\left(4^{k}+1\leq u\leq 4^{k}+\lambda \Lambda \right) \end{array}\right.$$

The former $4^{k}$elements are nucleotide composition features, which can reflect local or short-range sequence-order information. The latter λΛ factors are pseudo nucleotide composition features corresponding to global or long-range effect. In equation (2), $f_{i}^{k-tuple}$ represents the normalized frequency of occurrence of the i-th k-tuple nucleotides in the sample sequence. The weight factor ω can adjust the effects of nucleotide composition and local structural properties of DNA. And $\tau_{j}$ indicates the m-tier correlation factor and is formulated with the form of equation (3), the value of which corresponds to the sequence-order correlation between all the m-tier contiguous k-tuple nucleotide component along a promoter/non-promoter sequence.


(3) $$\left\{\;\begin{array}{c} \;\tau_{1}=\frac{1}{L-k}\sum_{i=1}^{L-k}J_{i,i+1}^{1}\;\\ \tau_{2}=\frac{1}{L-k}\sum_{i=1}^{L-k\;}J_{i,i+1}^{2}\;\\ \ldots \ldots \\ \tau_{\Lambda }=\frac{1}{L-k}\sum_{i=1}^{L-k}J_{i,i+1}^{\Lambda }\;\lambda <\left(L-k\right)\;\\ \ldots \ldots \\ \tau_{\lambda \Lambda -1}=\frac{1}{L-k-\lambda +1}\sum_{i=1}^{L-k-\lambda +1}J_{i,i+1}^{\lambda \Lambda -1}\;\\ \tau_{\lambda \Lambda }=\frac{1}{L-k-\lambda +1}\sum_{i=1}^{L-k-\lambda +1}J_{i,i+1}^{\lambda \Lambda }\; \end{array}\right.$$

where


(4) $$\{\begin{array}{c} \;J_{i,i+m}^{\xi }=H_{\xi }\left(R_{i}R_{i+1}\right)\cdot H_{\xi }\left(R_{i+m}R_{i+m+1}\right)\;\\ \xi =1,2,\cdot \cdot \cdot ,\Lambda ;m=1,2,\cdot \cdot \cdot ,\lambda ;i=1,2,\cdot \cdot \cdot ,L-\lambda -1 \end{array}$$

where $H_{\xi }\left(R_{i}R_{i+1}\right)$ is the standardized value of the ξ-th DNA local structural properties for the dinucleotide $R_{i}R_{i+1}$ at position i. The original values of these physicochemical properties are provided by Goñi et al. (2008) and the standardization approach are the same as previously described in iProEP. In addition, the processes of Position-Correlation Scoring Matrix (PCSM) construction and PCSF feature transformation and selection are directly referring to the *E. coli* model in iProEP.

### 2.3. mRMR

mRMR is a well-known feature selection method and has been used in many computational and biological applications (Zulfiqar et al., 2021; Su et al., 2023). The density functions are described as ‘*i*’ and ‘*y*’ and their corresponding probabilities are $P\left(i\right)$ and $P\left(y\right)$. The common information between these two functions can be demarcated as


(5) $$Z_{\min}\left(M_{i},M_{y}\right)=\underset{i\in Z}{\sum }\underset{y\in Y}{\sum }P\left(Mi,My\right)\log\frac{P\left(i,y\right)}{P\left(i\right),P\left(y\right)}$$

If the target is $J_{i}\;$then calculating the mutual information in relation to the target and can be defined as


(6) $$Z_{\max}\left(M_{i},J_{i}\right)=\underset{Mi\in Z}{\sum }\underset{Ji\in i}{\sum }P\left(Mi,Ji\right)\log\frac{P\left(Mi,Ji\right)}{P\left(Mi\right),P\left(Ji\right)}$$

$\text{So},\text{calculating the }\text{mRMR }\text{as }\left(M_{i}\right)$


(7) $$\text{mRMR}\left(M_{i}\right)=\frac{Z_{\max}\left(M_{i},J_{i}\right)}{Z_{\min}\left(M_{i},f_{y}\right)}$$

### 2.4. Machine learning classifiers

SVM is a well-known classifier and has been utilized in many bioinformatics and computational biology related tools (Basith et al., 2021; Arif et al., 2022; Basith et al., 2022; Bupi et al., 2023; Dao et al., 2023). It is typically used to perform binary classification. Ada boost (AB) is another famous classifier (Wang et al., 2021). The main idea of AB is to set the classifiers weights and trained the data in each and every iteration. Naïve Bayes (NB) classifier has been widely used in bioinformatics due to its simplicity (Naseer et al., 2022; Zulfiqar et al., 2022). This classification method totally depends on the Bayes theorems. Random Forest (RF) is a collective knowledge algorithm and broadly used in bioinformatics (Zhu et al., 2022; Zhang et al., 2023). The main idea of this is to unite multiple weak classifiers and outcome generated on the basis of voting (Zulfiqar et al., 2023). The brief description is clearly described in (Zulfiqar et al., 2021). The k-nearest neighbor (KNN) is a non-parametric and supervised learning classifier, which uses vicinity to make classifications about the grouping of an individual data point. Logistic Regression (LR) is a classification algorithm and used when the value of the target variable is categorical in nature (Yang et al., 2021). We have executed these algorithms in Weka version 3.8.4. by using the default values.

### 2.5. Evaluation metrics

Accuracy, sensitivity, specificity (Cao et al., 2017; Tang et al., 2022; Yang et al., 2022; Zhang et al., 2022; Chen et al., 2023) were utilized to evaluate the performance of the prediction model and termed as


(8) $$\{\begin{array}{c} Sn=\frac{tp}{tp+fn}\;\\ Sp=\frac{tn}{tn+fp}\;\\ Acc=\frac{tp+tn}{tp+fp+tn+fn}\; \end{array}$$

where ‘*tp*’ represents the correctly predicted promoter sequences and ‘*fp*’ shows the non-promoter sequences classified as promoter sequence. And the other hand, ‘*tn*’ characterizes the correctly recognized non-promotor sequences and ‘*fn*’ exhibit the promoter sequences which were classified as non-promoter sequence.

## 3. Results and discussion

In the fields of statistical analysis and machine learning (ML) prediction, cross-validation (CV) strategy has been widely utilized to evaluate the prediction performance of ML models (Hasan et al., 2022; Shoombuatong et al., 2022; Xiao et al., 2022; Yu et al., 2022; Zhang et al., 2022). In this work, *5*-fold CV technique was used in the processes of PseKNC parameter optimization and optimal feature subset selection and *10*-fold CV technique was used to assess the performance of the six machine learning methods. In *n*-fold CV, the benchmark dataset was randomly divided into *n* groups with equal size. Each group was individually tested on the model which was trained with the remaining *n*-1 groups. According to this, the *n*-fold CV method was performed *n* times, and the final evaluation result was the average prediction performance of the *n* models.

We constructed a computational model on the basis of sequence features to recognize promoter sequences in *K. aerogenes*. Based on the definition of pseudo nucleotide characteristics, we debugged the parameters k, λ, and ω according to the following range to determine the optimal combination of *k*-mer nucleotide composition information and long-range sequence order information.,


$$\{\begin{array}{c} k\in \left[2,5\right],\;\;step=1\;\\ \lambda \in \left[1,30\right],\;\;step=1\;\\ \omega \in \left[0.1,1\right],\;\;step=0.1\; \end{array}$$

**Figure 2:**
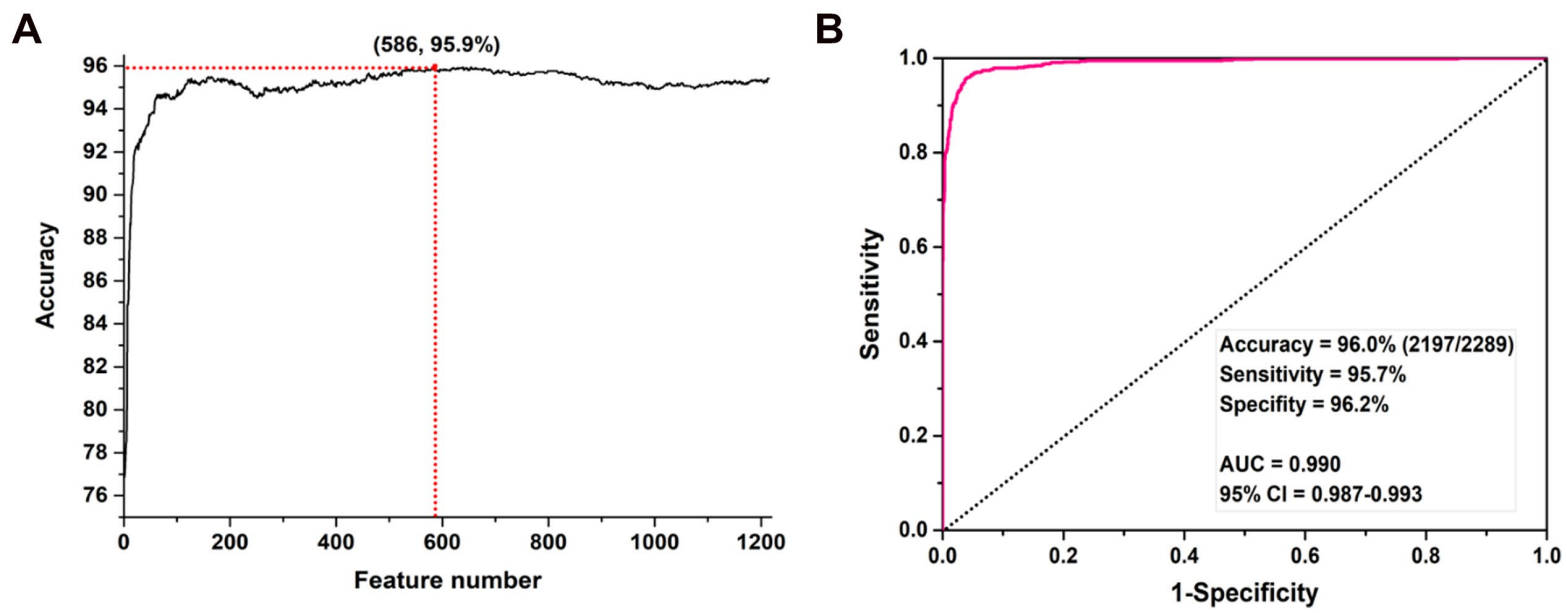
The prediction accuracies of SVM models constructed with different numbers of features. **(A)** IFS process for feature selection and **(B)** ROC curve based on the optimal features.

Based on the feature set generated by each combination and the LIBSVM algorithm, we can construct promoter prediction models and evaluate their accuracies using a 5-fold CV method. The final determined values of *k*, *λ*, and *ω* were 5, 29, and 0.1, respective. The original vector contains 1,198 features which could produce the prediction accuracy of 88.0%. Then, 17 positional correlation scoring features were calculated based on the most conserved sites in the promoter sequence of the 3-mer nucleotide fragment. After integrating two types of features, the mRMR algorithm was applied to sort all features, and an incremental feature selection (IFS) method was applied to eliminate redundant information to obtain the optimal feature subset for improving the accuracy of the promoter classifier. In the process of IFS, we also used a 5-fold CV method to evaluate the promoter prediction accuracy of each classifier, as shown in Figure 2A. As shown in the figure, the model constructed based on the first 586 features has the highest prediction accuracy of 95.9%.

After determining the optimal subset of features, we further evaluated its promoter prediction ability using a 10-fold CV method for determining the parameters *c* and *γ* in SVM, where *c* ∈ [2^−5^, 2^15^] with a step of 2, *γ*∈ [2^3^,2^−15^] with a step size of 2^−1^. The final optimal values of *c* and *γ* are 2 and 2^−3^, respectively. The optimal SVM model could produce the best performance with the accuracy of 96.0%, sensitivity of 95.7%, and specificity of 96.2%. The area under the ROC curve (AUC) was 0.990 with 95% confidence interval (CI): 0.987–0.993 (as shown in Figure 2B).

**Figure 3:**
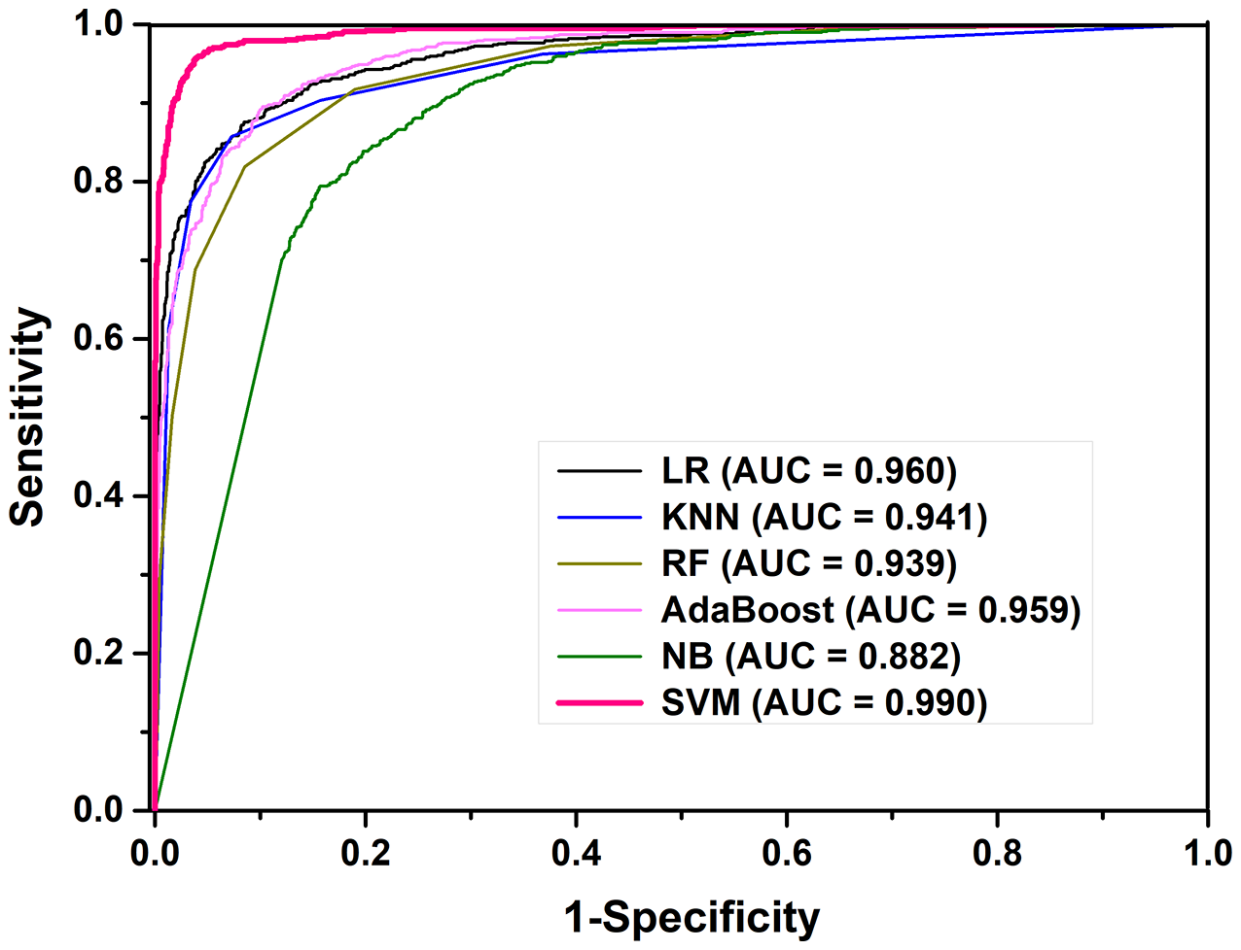
The ROC curves for different machine learning models.

In order to evaluate the performance of this SVM prediction model, we also constructed five models based on LR, KNN, RF, AB and NB for *K. aerogenes* promoter recognition by using the same optimal features. The 10-fold CV results showed that the AUC values of the LR, KNN, RF, and AB models were 0.960, 0.941, 0.939, and 0.959, respectively, as shown in Figure 3. We observed that the sensitivity of the RF model was poor (68.8%), while the overall predictive performance of the NB model was the weakest, with accuracy and AUC values of 81.3% and 0.882 (Table 1). The accuracy of SVM-based model was 96.0% which was 5.6–14.7% higher than the other five classifiers. Overall, identifying *K. aerogenes* promoter sequences based on optimal pseudo nucleotide features and positional correlation scoring features is effective, and the model constructed based on SVM algorithm has the best predictive performance.

**Table 1:** The prediction performance of different machine learning models based on 10-fold cross-validation.

Method	Sn (%)	Sp (%)	Acc (%)	AUC
LR	85.1	93.1	90.4	0.960
KNN	85.7	92.7	90.4	0.941
RF	68.8	96.2	87.1	0.939
AB	84	92.9	89.9	0.959
NB	83.9	79.9	81.3	0.882
**SVM**	**95.7**	**96.2**	**96.0**	**0.990**

Note: The *K. aerogenes* promoter prediction model constructed with SVM classifier produces the highest accuracy, sensitivity, specificity and AUC, which is the finally determined model.

## 4. Conclusion

Promoters play an important role in the initiation of transcription, because they are located upstream of genes. RNA polymerase and a quantity of transcription factors bind to promoter to start the transcription. Therefore, studying promoters is crucial for studying gene expression regulation. In this study, we proposed an SVM-based model to identify promoter sequences in *K. aerogenes*. In the proposed model, sequences were encoded using PseKNC and PCSF and then optimized with mRMR and SVM-based algorithm on 5-fold CV. Then, these optimized features were inputted into SVM-based classifier using 10-fold CV and achieved the best model. The results show that our model can predict promoters accurately, suggesting that our feature extraction and selection methods are able to capture the important sequence features. In the future, we will develop more suitable and robust models for more prokaryotic species.

## Data availability statement

Publicly available datasets were analyzed in this study. This data can be found at: http://lin-group.cn/database/ppd/.

## Author contributions

YL, HZ, and HL project design and oversight, and manuscript writing and revision. MS and HZ sample collection and curation. YL, JZ, HZ, ML, and KY experiment conduction and data analysis. YL and ML table preparation. YL, MS, and CW result interpretation and discussion. YL and JZ funding acquisition. All authors contributed to the article and approved the submitted version.

## Funding

This research was funded by the grant from the National Natural Science Foundation of Sichuan Province (no. 2022NSFSC0058), the Sichuan Key Science and Technology Project (no. 2021ZDZX0009), the Research Program of Science and Technology at Universities of Inner Mongolia Autonomous Region under Grant NJZZ18381 and the National Natural Science Foundation of China (62262049).

## Conflict of interest

The authors declare that the research was conducted in the absence of any commercial or financial relationships that could be construed as a potential conflict of interest.

## Publisher’s note

All claims expressed in this article are solely those of the authors and do not necessarily represent those of their affiliated organizations, or those of the publisher, the editors and the reviewers. Any product that may be evaluated in this article, or claim that may be made by its manufacturer, is not guaranteed or endorsed by the publisher.

## Footnotes

^1^ http://lin-group.cn/server/iProEP/
